# Safeguarding maternal mental health in the perinatal period

**DOI:** 10.1016/j.eclinm.2024.102663

**Published:** 2024-05-17

**Authors:** eClinicalMedicine

April 29–May 5 marked Maternal Mental Health Awareness Week 2024, encompassing the international awareness day on May 1, with an aim to promote discussion about mental health in women who are pregnant or have given birth. The perinatal period, which includes pregnancy and the first year postpartum, is a time of great vulnerability for women, involving substantial physiological and lifestyle changes, where mental health is particularly at risk. Hence, perinatal mental health is a crucial but frequently overlooked and neglected element of maternal and infant care.

There is an alarming prevalence of maternal mental health disorders during the perinatal period worldwide. WHO estimates that approximately 10% of women who are pregnant and 13% of those who have just given birth will develop a mental health disorder. Moreover, these rates are higher (about 16% during pregnancy and 20% after childbirth) in low-income and middle-income countries (LMICs), where access to mental health care is limited, as shown by the presence of less than one mental health worker per 100,000 population in low-income countries (compared with about one per 2000 in high-income countries). While the most common maternal mental health disorder is postnatal depression, others include anxiety, postpartum psychosis, post-traumatic stress disorder, schizophrenia, and bipolar disorder. Suicide is a leading cause of maternal mortality, accounting for about 20% of postpartum deaths, showing the devastating effect that perinatal mental health issues can have.

Risk factors for mental health problems in the perinatal period include history of mental illness, biological factors, poverty, an absence of a social support network, racism and discrimination, migration, extreme stress, exposure to violence, emergency and conflict situations, natural disasters, and trauma. Women in marginalised communities are therefore at increased risk of having a mental health disorder during the perinatal period. Thus, addressing systemic inequalities and ensuring equitable access to health care and support services is fundamental to improving perinatal mental health. Additionally, social support and the role of family, partners, and friends can also be crucial to protect mental health during pregnancy and after childbirth.

There are numerous barriers that limit the detection and treatment of perinatal mental health disorders, such as paucity of screening, insufficient obstetric provider training, scarcity of mental health resources, and stigma. Stigma is a major challenge in addressing all mental health disorders, and this is particularly apparent in the context of motherhood, where additional societal expectations and demands are placed upon mothers, which can induce feelings of shame or guilt and further deteriorate maternal mental health. This stigma can lead to reluctance to seek help, as well as underreporting of disorders, and must be eradicated to create a safe and supportive environment where concerns can be shared. The COVID-19 pandemic also led to increased maternal anxiety and depression in the perinatal period, due to worries about vertical transmission of the virus, limited accessibility to appointments and antenatal care resources, and reduced social interaction and support. The long-term effects of the pandemic are still emerging, but it is important to anticipate and enaction plans to address the increased prevalence and complexity of symptoms, as well as the consequences of untreated maternal mental health problems.

Maternal mental health disorders can have profound and lasting effects on both mothers and infants if not treated. Mothers with depression might have difficulty in eating, sleeping, and other aspects of care for themselves and their babies, which can increase the risk of other health conditions. Additionally, prolonged or severe mental illness hampers mother–newborn attachment, breastfeeding, and infant care. Untreated maternal mental health disorders have been shown to be associated with adverse birth outcomes, such as stillbirth and preterm birth, and detrimental effects on infant development, including cognitive, behavioural, and psychomotor issues, which in turn increases the risk of neuropsychiatric disorders in later life.

In *eClinicalMedicine*, Elysia Davis and colleagues report their findings of a post-hoc analysis of a randomised controlled trial that found brief interpersonal psychotherapy significantly reduced prenatal depression symptoms compared with usual care. In the post-hoc analysis, the authors found that reducing maternal depression across pregnancy with interpersonal psychotherapy led to lengthened gestation and an increase in the number of babies born at full term (odds ratio 1.54, 95% CI 1.10–2.16 for birth at ≥39 gestational weeks; p = 0.01). Also recently published in our pages, a large, multinational cohort study reported by Claudia Bruno and colleagues, found no significant increase in risk of child neurodevelopmental disorders or learning difficulties after prenatal exposure to antipsychotics, which might provide some reassurance for women and clinicians regarding the use of pharmacological interventions during this period.

Identification, prevention, treatment, and raising awareness of maternal mental health disorders in the perinatal period should be a priority. Comprehensive screening programmes are vital for early identification and intervention to address maternal mental health disorders during the perinatal period. Routine screening should be a part of standard prenatal and postnatal care to ensure that women receive appropriate support as early as possible. Furthermore, all women should have access to comprehensive perinatal mental health care, involving both pharmacological and non-pharmacological interventions, such as community support groups and cognitive therapy. Two systems-level interventions in the USA, the Massachusetts Child Psychiatry Access Program for Moms (MCPAP for Moms) and the Program In Support of Moms (PRISM), were recently shown to be equally effective in improving perinatal depression. Such resources are scarce in LMICs, and novel viable interventions and strategies for improvement are still needed globally. Moreover, mental health services should be integrated into the frequent general health care visits during pregnancy and after childbirth to improve access and reduce stigma and to provide referral and support.

Safeguarding maternal mental health during the perinatal period is a public health challenge of paramount importance. Health-care professionals, policy makers, and communities must act together to prioritise maternal mental health so that all women receive the care that they require during this vulnerable time. Through addressing the risk factors for maternal mental health disorders, dismantling the barriers to detection and accessing support services, and implementing interventions earlier and more effectively, the future health of both mother and child can be greatly improved.

